# Influence of the acute alcoholism on the phagocytic function of the mononuclear phagocytic system

**Published:** 2011-11-24

**Authors:** KR Sabino, A Petroianu, LR Alberti

**Affiliations:** * Santa Casa de Belo Horizonte; ** Federal University of Minas Gerais, Brazil; *** Santa Casa de Belo Horizonte, MG and Federal University of Minas Gerais, Brazil

**Keywords:** Alcoholism, Mononuclear phagocyte system, Phagocytosis, Sulphur colloid, Ethanol

## Abstract

**Rationale:**Alcoholics are more likely to have infections, mainly in the respiratory system. Alcohol seems to inhibit the immune system. Despite the extensive literature related to alcoholism, data related to the immune system are still not conclusive.

**Objective:** The purpose of this study was to verify the influence of acute alcohol intake on colloid distribution in the organs of the mononuclear phagocyte system.

**Methods and Results:** Thirteen male Swiss mice were divided into two groups: Group 1 (n = 5) – control, and Group 2 (n = 8) – animals that received 0.5 ml ethanol 50%, 30 minutes before the experiment. Colloidal sulphur labeled with ⁸⁸mTc was used to evaluate colloid distribution in the liver, spleen and lungs. Colloid clearance was assessed as well. A gamma camera was used to measure the radioactivity of these organs and of a blood clot. No difference was found in the presence of colloid in the organs of both groups. The liver showed the highest phagocytic intake, followed by the spleen and lungs (p = 0.021 for Group 1 and p = 0.003 for Group 2). A minimum amount of radiation remained in the blood of both groups.

**Discussion:** According to the experiential conditions of this work, acute ingestion of alcohol did not interfere with the phagocytic function of the mononuclear phagocyte system in mice.

## Introduction

Only in the 19th century, alcoholism started being scientifically accepted as a disease in its broader concept, as a physical and mental entity [**1**]. This disorder is one of the major public health problems in the world. According to the World Health Organization (WHO), 20% of population in developing countries is alcoholic. The excessive consumption of this substance is directly or indirectly responsible for 75% of psychiatric hospitalizations and 50% of hospitalizations in internal medicine wards. It also represents a significant socioeconomic problem, since the alcoholic individual shows low productivity at work as well as a high number of absences as a result of recurrent hospitalizations [**1-2**].

The metabolic effects of alcohol are well-known, including decreased synthesis of albumin, in addition to decreased serum concentration of magnesium, calcium and phosphate. It is also responsible for blocking gliconeogenesis, with consequent hypoglycemia, increased lactate, ketone and oxygen consumption. Due to its direct toxic action, it causes impaired erythropoiesis and granulocyte formation and, due to its indirect action, reduced absorption of folic acid and inadequate red blood cell production [**2-6**].

Infectious complications resulting from alcohol consumption have been described in the literature since 1785 [**7**]. Alcoholics show higher incidence of sepsis, mainly of respiratory origin [**3**]. The immune response may be compromised in several stages and immunodepression is caused by both abnormalities in the primary defense mechanisms and in the cellular and humoral immunity. These disorders occur in the mucociliary activity of the respiratory epithelium, in cough reflex, IgA release by the mucosa and in the decreased number of lymphocytes, especially natural killer cells. Granulocyte chemotaxis and secretion of tumor necrosis factor are also diminished [**8-9**].

Complications secondary to the alcoholism associated with nutritional deficiencies and social imbalances contribute to the organic frailty and predispose the individual to infections [**1-2**]. Despite the existing studies suggesting the interference of alcoholism in the mononuclear phagocyte system (MPS), the effect, after acute ingestion of alcohol in the phagocytic function in the liver, spleen and lungs, as well as in blood clearance is still controversial. 

The purpose of this study was to verify the influence of acute alcoholism in the phagocytic function of liver, spleen and lungs, and its effects on blood clearance.

## Methods

This study was carried out in accordance with the guidelines of the International Regulations for Animal Protection and was approved by the Animal Experimentation Ethics Committee of Federal University of Minas Gerais [**10-11**].

A total of 13 male Swiss mice with mean weight of 30 ± 5 g were distributed into two groups: Group 1 (n = 5) – control and Group 2 (n = 8), that received 0.5 ml aqueous solution of 50% ethanol via orogastric catheter. After 30 minutes, all animals in both groups underwent general anesthesia with injection of pentabarbiturate at a dose of 3.5 mg/animal (90 mg/kg) and fentanyl citrate 2.5 μg/animal (60 μg/kg) through intraperitoneal route. Afterwards, the right internal jugular vein was dissected and a technetium-labeled sulphur colloid solution was injected, corresponding to 3 mCi (110 MBq) in a dose of 1 ml/ kg of body weight [**12-17**]. One hour after the colloid injection, the mice were killed with a lethal dose of pentabarbiturate. Subsequently, fragments measuring 1 cm were obtained from the right hepatic lobe, middle portion of the spleen and lower lobe of left lung. A blood clot was also obtained from a section of caudal vena cava.

Fragments from each organ and the blood clot were placed in plastic cups and weighed. Radioactivity contained in each sample was determined by placing the cups with samples in a collimator center of a scintigraph (Siemens, model Orbiter) for measurement of gamma rays (gamma camera). Radiation was calculated per gram of tissue [**12-17**]. Two empty cups had been previously placed inside the gamma camera collimator to assure complete absence of radioactivity. To facilitate data interpretation, the total sum of radiation in all samples was assigned a value of "1". Based on this result, radioactivity of each segment was converted to a value proportional to one.

Mean values of samples from different groups were compared by the Student’s t test. Significant differences were those corresponding to a p value of < 0.05.

## Results

All animals remained without apparent abnormalities during the experiment. (**Table 1**) shows the percentage values of radioactivity records in the samples studied as well as in the blood clot.

**Table 1:** Proportional values (mean ± standard error of the mean) of radioactivity records in control mice (Group 1) and mice acutely alcoholized (Group 2) in samples of phagocyte mononuclear system and blood clot.

SAMPLES	GROUP 1	GROUP 2	p
Liver	0.54 + 0.9 *¹	0.52 + 0.18 *²	0.435
Spleen	0.22 + 0.6	0.24 + 0.11	0.237
Lung	0.17 + 0.3	0.19 + 0.4	0.142
Blot	0.01 + 0.00 *¹	0.03 + 0.02 *²	0.184
*¹: comparison between radioactivity of liver and clot (p = 0.021) *²: comparison between radioactivity of liver and clot (p = 0.003)

No differences were observed in colloid uptake between Group 1 and 2. Both groups showed increased phagocytic activity in the liver, followed by the spleen and lungs. The blood clot retained a minimum amount of radiation.

## Discussion

Chronic alcoholism depresses the production of polymorphonuclear cells (PMN) by the bone marrow. In acute infection, granulocytopenia that occurs in alcoholics may be just the suppression caused by alcohol [**7**].

Acute alcoholic intoxication impairs the mobilization of PMN to the inflammation sites, thus limiting the host capacity to control a local bacterial infection. This reduction in the release of PMN does not happen in chronic alcoholics with subtoxic alcohol levels or in patients with cirrhosis. Phagocytosis and granulocyte bactericidal activity are not inhibited by alcohol [**1**],[**7**],[**18-19**],[**20**].

Alcohol also decreases cellular immunity. In alcoholic individuals, the number of T-lymphocytes is lower, as well as their ability to suffer blastic transformation through mitogenic stimulation. It is also associated with a deficient response to antigen skin tests to which the patient has been previously sensitized, accompanied by failure of immune response to new antigens. Experimentally, incubation of lymphocytes from normal individuals in a culture medium containing alcohol results in decreased cytotoxic activity and in migratory movement of these lymphocytes. Therefore, alcohol interferes with both in vivo and in vitro cellular immunity. Malnutrition seems to corroborate with the immunodepression caused by alcohol [**7-8,18**].

According to some authors, alcohol decreases in vitro phagocytosis of the MPS in concentrations higher than 6.4 g/l. These values are higher than those found in the clinical practice, which are around 2 g/l [**2**]. However, in this study, there were no records of significant abnormalities in the uptake of radio labeled colloid by the different organs of the MPS and in blood clearance.

Liver of both normal and alcohol-treated animals showed higher uptake of colloidal sulphur. We believe that the predominant clearance in the liver is due to the high blood flow associated with a higher number of macrophages in this organ. Some authors have reported decreased phagocytic activity in the Kupffer cells of alcoholics [**8**],[**19**]. However, during the short follow-up period of this study, no reduction in colloid distribution was seen in the MPS.

## Conclusion

In this study, acute administration of ethanol did not influence the pattern of colloid distribution in the MPS or its blood clearance.
